# Optical Fiber Power Meter Comparison Between NIST and NIM*

**DOI:** 10.6028/jres.115.029

**Published:** 2010-12-01

**Authors:** I. Vayshenker, D. J. Livigni, X. Li, J. H. Lehman, J. Li, L. M. Xiong, Z. X. Zhang

**Affiliations:** Optoelectronics Division, National Institute of Standards and Technology, Boulder, CO 80305, USA; National Institute of Metrology, No. 18, Beisanhuan Donglu Rd., Chaoyang Dist., Beijing 100013, China

**Keywords:** international comparison, optical fiber, optical power

## Abstract

We describe the results of a comparison of reference standards between the National Institute of Standards and Technology (NIST-USA) and National Institute of Metrology (NIM-China). We report optical fiber-based power measurements at nominal wavelengths of 1310 nm and 1550 nm. We compare the laboratories’ reference standards by means of a commercial optical power meter. Measurement results showed the largest difference of less than 2.6 parts in 10^3^, which is within the combined standard (*k* = 1) uncertainty for the laboratories’ reference standards.

## 1. Introduction

In our previous work [1–7, we reported the results of international comparisons of reference standards used in the calibration of optical fiber power meters (OFPMs). Those reports describe the results that were obtained by use of open laser beams [1, 4, 7] and optical fiber cables [2–7] at 1310 nm and 1550 nm. We also compared internal NIST laser and OFPM standards at several laser wavelengths in the visible and near infrared [8]. In this paper, the reference standards maintained by the two national laboratories (NIST and NIM) were compared at nominal wavelengths of 1310 nm and 1550 nm by launching optical power from a reference optical fiber.

For OFPM measurements, the primary standard of both NIST [9] and NIM [10] are cryogenic radiometers that have uncertainties of 2 parts in 10^4^ (*k* = 1). Typically, reference standards are calibrated against the primary standards by use of open (free-field) collimated beams, but are generally used with divergent beams of laser light exiting an optical fiber. Most primary standards are designed to be used with open beams rather than divergent beams from an optical fiber.

For the comparison of reference standards we used a commercial OFPM, which was calibrated at both national laboratories against their reference standards at nominal wavelengths of 1310 nm and 1550 nm. This transfer standard is also referred as Device Under Test (DUT). The same reference fiber cable was used by the laboratories, which employed a direct substitution method for their measurements.

## 2. NIST and NIM Measurement Systems

**Fig. 1:**
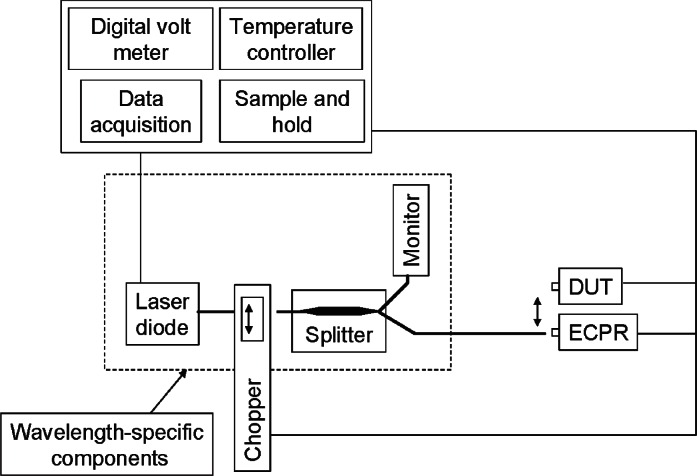
NIST optical power measurement system.

The NIST measurement system, described in detail in [11] and depicted in Fig. 1, consists of fiber-pigtailed laser sources at wavelengths of 1306.5 nm and 1549.6 nm (all center wavelengths in this paper are based on refractive index in vacuum), a reference optical fiber cable, and a positioning stage (see double-headed arrow) for comparing the NIST reference and transfer standards. The output of each laser source is transmitted through a fiber to a fiber splitter from which about 1 % of the power travels to a monitor detector. The remaining 99 % of the power is transmitted to the reference optical fiber cable that is used in the comparison.

The NIST reference standard is an electrically calibrated pyroelectric radiometer (ECPR) that had been previously calibrated against a primary standard, the NIST Laser Optimized Cryogenic Radiometer (LOCR). The ECPR consists of a thermal detector that is covered with gold black coating. The response of the ECPR does not depend on the wavelength of the incident radiation over the wavelength region of 1300 nm–1550 nm [12].

The NIM measurement system is similar to the NIST system. It consists of fiber-pigtailed laser sources at wavelengths of 1301.2 nm and 1549.2 nm, reference optical fiber cable, and a positioning stage for comparing the NIM reference and transfer standards. The NIM reference standard, the Electrically Calibrated Absolute Radiometer (ECAR) is a thermal device that had been calibrated against the NIM cryogenic radiometer.

## 3. Results of the Comparison

The NIST and NIM reference standards were compared by means of a commercial transfer standard and a reference optical fiber cable at nominal wavelengths of 1310 nm and 1550 nm. The power was approximately 100 μW (−10 dBm). The standard uncertainties for the optical power measurements were evaluated in accordance with ISO document standards [13].

Both laboratories used the same reference optical fiber cable. At NIST eight measurement runs were taken with relative standard deviation of 1 × 10^−4^ at a wavelength of 1310 nm, and six measurement runs were taken with relative standard deviation of 2 × 10^−4^ at a wavelength of 1550 nm. At NIM, nine measurement runs were taken with a relative standard deviation of 0.7 × 10^−3^ at 1310 nm and a relative standard deviation of 1.5 × 10^−3^ at 1550 nm. The results of the comparison are given in Table 1.

At 1310 nm the relative difference between the NIST and NIM results was 2.2 parts in 10^3^, and at 1550 nm the relative difference was 2.6 parts in 10^3^ (the plus sign for both relative differences indicates that the NIM reference standard read lower than NIST’s). The NIST standard uncertainty was 1.9 parts in 10^3^ at 1310 nm and 2.4 parts in 10^3^ at 1550 nm, while that of NIM was 3.5 parts in 10^3^ at both wavelengths.

Table 1 provides values of relative combined standard uncertainty for NIST and NIM. These values are calculated by taking a square root of the sum of the squares of each laboratory standard uncertainty. The observed interlaboratory differences are less than the relative combined standard (*k* = 1) uncertainties for the laboratories’ reference standards.

## 4. Conclusion

This optical power meter comparison shows a good agreement between NIST and NIM measurements. The purpose of this work is to verify a consistency in measurements of optical power in the area of optical telecommunications.

## Figures and Tables

**Table 1:** Results of NIST and NIM comparison

Source wavelength (nm)	difference (%)	NIM standard uncertainty (%)	NIST standard uncertainty (%)	combined standard uncertainty (%)
1310	0.22	0.35	0.19	0.40
1550	0.26	0.35	0.24	0.43
